# Noninvasive quantification of SIRT1 expression–activity and pharmacologic inhibition in a rat model of intracerebral glioma using 2-[^18^F]BzAHA PET/CT/MRI

**DOI:** 10.1093/noajnl/vdaa006

**Published:** 2020-01-16

**Authors:** Maxwell T Laws, Robin E Bonomi, David J Gelovani, Jeremy Llaniguez, Xin Lu, Thomas Mangner, Juri G Gelovani

**Affiliations:** 1 Department of Biomedical Engineering, College of Engineering and School of Medicine, Wayne State University, Detroit, Michigan, USA; 2 Positron Emission Tomography Center, Wayne State University, Detroit, Michigan, USA; 3 Department of Oncology, Wayne State University School of Medicine, Detroit, Michigan, USA; 4 Department of Neurosurgery, Wayne State University School of Medicine, Detroit, Michigan, USA; 5 Molecular Imaging Program, Karmanos Cancer Institute, Wayne State University School of Medicine, Detroit, Michigan, USA

**Keywords:** epigenetics, EX-527, glioma, molecular imaging, SIRT1

## Abstract

**Background:**

Several studies demonstrated that glioblastoma multiforme progression and recurrence is linked to epigenetic regulatory mechanisms. Sirtuin 1 (SIRT1) plays an important role in glioma progression, invasion, and treatment response and is a potential therapeutic target. The aim of this study is to test the feasibility of 2-[^18^F]BzAHA for quantitative imaging of SIRT1 expression–activity and monitoring pharmacologic inhibition in a rat model of intracerebral glioma.

**Methods:**

Sprague Dawley rats bearing 9L (*N* = 12) intracerebral gliomas were injected with 2-[^18^F]BzAHA (300–500 µCi/animal i.v.) and dynamic positron-emission tomography (PET) imaging was performed for 60 min. Then, SIRT1 expression in 9L tumors (*N* = 6) was studied by immunofluorescence microscopy (IF). Two days later, rats with 9L gliomas were treated either with SIRT1 specific inhibitor EX-527 (5 mg/kg, i.p.; *N* = 3) or with histone deacetylases class IIa specific inhibitor MC1568 (30 mg/kg, i.p.; *N* = 3) and 30 min later were injected i.v. with 2-[^18^F]BzAHA. PET-computerized tomography-magnetic resonance (PET/CT/MR) images acquired after EX-527 and MC1568 treatments were co-registered with baseline images.

**Results:**

Standard uptake values (SUVs) of 2-[^18^F]BzAHA in 9L tumors measured at 20 min post-radiotracer administration were 1.11 ± 0.058 and had a tumor-to-brainstem SUV ratio of 2.73 ± 0.141. IF of 9L gliomas revealed heterogeneous upregulation of SIRT1, especially in hypoxic and peri-necrotic regions. Significant reduction in 2-[^18^F]BzAHA SUV and distribution volume in 9L tumors was observed after administration of EX-527, but not MC1568.

**Conclusions:**

PET/CT/MRI with 2-[^18^F]BzAHA can facilitate studies to elucidate the roles of SIRT1 in gliomagenesis and progression, as well as to optimize therapeutic doses of novel SIRT1 inhibitors.

**Key Points**

1. PET/CT/MRI with 2-[18F]BzAHA is effective for noninvasive quantification of SIRT1 expression-activity in 9L gliomas in rats.
2. PET/CT/MRI with 2-[18F]BzAHA can help in monitoring the pharmacodynamics of SIRT1 inhibitors.
3. PET/CT/MRI with 2-[18F]BzAHA can be readily translated into the clinic for the selection of patients for SIRT1-targeted therapies.

**Importance of the Study**

SIRT1 plays an important role in gliomagenesis, progression, and responses to temozolomide and radiotherapy. This study aimed to assess the efficacy of PET/CT/MRI with 2-[^18^F]BzAHA for noninvasive and quantitative imaging of SIRT1 expression–activity in the 9L intracerebral glioma model in rats. Since PET/CT/MRI is a clinical imaging modality, imaging SIRT1 expression–activity with 2-[^18^F]BzAHA can be readily translated into the clinic. PET/CT/MRI with 2-[^18^F]BzAHA could be potentially utilized for selection of glioma patients with high levels of SIRT1 expression–activity levels in tumor tissue that may benefit from SIRT1-targeted pharmacologic and genetic therapies, including miR mimics, for monitoring the pharmacodynamics and dose-dependency of SIRT1 inhibition and prediction of treatment response.

Glioblastoma multiforme (GBM) is the most common type of malignant primary brain tumors, comprising 53.8% of all malignant primary brain tumors, with the annual incidence of about 3 cases per 100 000 people per year and the mean survival time of just 15 months after diagnosis.^1^ In the last decade, progress toward treatments with improved overall survival and prolonged time to progression has largely stalled. Therefore, research in molecular genetics of gliomas has come to the forefront for the development of novel, more effective therapies. Several studies demonstrated that GBM progression and recurrence is linked to epigenetic regulatory mechanisms including mutations in IDH1/IDH2 genes, epigenetic modifying enzymes, histone deacetylases (HDACs), histone methyltransferases, DNA methyltransferases, and various DNA demethylases.^2^ These epigenetic regulators contribute to malignant transformation and progression by influencing the spectrum and magnitude of gene expression, DNA repair, cell cycle, stability and function of non-histone proteins involved in glioma cell signaling and regulation of metabolism, apoptosis, and senescence.^3–5^ In particular, HDACs cleave an acetyl moiety from the Ɛ-amino terminus of lysine residues on histone core and non-histone proteins. Among 4 classes of HDACs, class III HDACs are homologous to yeast Sir2 protein, known as “silent information regulators” or “sirtuins,” and include 7 isotypes (SIRTs1–7).^6^ SIRTs differ from other HDAC classes in that their catalytic activities are NAD^+^-dependent, whereas the other HDACs are Zn-dependent. Many SIRTs are involved in the regulation of protein stability and functions outside of the nucleus and cleave other moieties aside from acetyl, such as malonyl, succinyl, glutaryl, lipoyl, palmitoyl, and others.^7^

In particular, SIRT1 has been implicated in a variety of disease processes, including diabetes, cardiovascular disease, and neurodegeneration.^8^ Also, SIRT1 is involved in the pathogenesis of multiple malignancies, including hematologic,^9^ oral,^10^ pancreatic,^11^ liver,^12^ lungs,^13^ breast,^14^ and brain.^15^ In the nucleus, SIRT1 deacetylates several histone lysines, including H1K26,^16^ H3K4, H3K9, and H4K16.^17^ Deacetylated histone protein tails bind with higher affinity to adjacent DNA, hindering the binding of transcription complexes and effectively “silencing” gene expression.^18^ Global loss of acH4K16 has been described as a hallmark of cancer in humans and associated with early stages of tumor formation.^19^ SIRT1 shuttles from the nucleus into the cytoplasm,^20,21^ where it deacetylates and regulates the stability and function of several non-histone proteins, including p53, p73, FOXO, E2F1, NF-κB, and others.^22–24^ SIRT1 deacetylates and stabilizes HIF-1α,^25^ thereby increasing expression of VEGF, GLUT1, and MMP2.^25^ Increased SIRT1 expression within tumor cells promotes cell survival, proliferation, and angiogenesis in conditions of oxidative and metabolic stress through dysregulation of apoptotic pathways.^26–32^ Genetic and pharmacologic inhibition of SIRT1 activity arrests tumor growth and restores proper apoptotic signaling.^32–35^

SIRT1 is required for oncogenic transformation of neural stem cells and for the survival of “cancer cells with neural stemness” in a p53-dependent manner.^15^ Upregulation of SIRT1/PGC-1α is associated with increased chemo- and radioresistance of glioblastoma stem cell clones,^36^ while the shRNA-induced knockdown of SIRT1 expression enhances the effectiveness of radiotherapy by inhibiting tumor growth in CD133+ GBM xenografts in mice.^37^ SIRT1 suppression by miR-320 results in inhibition of forkhead box protein M1 and enhancement of radiosensitivity of U251 and U87 glioma cells in vitro.^38^ Correspondingly, the upregulation of miR-181a promotes glioma temozolomide (TMZ) sensitivity.^39^ Also, the downregulation of SIRT1 through upregulation of miR-34a provoked the expression of senescence-related genes p53, Cdkn1a, and Cdkn2c. Furthermore, induction of miR-34a and subsequent SIRT1 inhibition induced DNA damage, shortened telomere length, and impaired telomerase activity, functions that could synergize with existing chemoradiation therapies.^40^ Given the results of these prior studies, SIRT1 has emerged as a promising therapeutic target to aid in the treatment of numerous cancers including GBM.

Molecular imaging with PET/CT/MRI is a promising approach for the noninvasive, repetitive, visualization of expression–activity of HDAC enzymes, including SIRT1. Previously, we developed and validated novel radiotracers for PET imaging of HDACs class IIa, termed [^18^F]FAHA^41,42^ and [^18^F]TFAHA.^43,44^ More recently, we reported 2 novel radiotracers—2-[^18^F]BzAHA^45^ and [^18^F]DDAHA^46^ for PET imaging of SIRT1 and SIRT2, respectively. In the current study, we demonstrate the efficacy of PET/CT/MRI with 2-[^18^F]BzAHA for imaging the expression–activity of SIRT1 and for noninvasive monitoring of EX-527 induced inhibition of SIRT1 activity in 9L intracerebral glioma models in rats.

## Materials and Methods

### In Vivo Imaging Studies in Rats

Animal care and use procedures were carried out in accordance with protocols written under the guidelines of the National Institutes of Health Guide for the Care and Use of Laboratory Animals and approved by the Institutional Animal Care and Use Committee of Wayne State University (protocol #17-06-283).

### Intracerebral Tumor Implantation

9L rat gliosarcoma cells were obtained from the American Tissue Culture Collection (ATCC) and propagated in tissue culture treated T75 flasks (Corning). The 9L cells were cultured in DMEM supplemented with 10% FBS and 1% penicillin/streptomycin. For intracerebral (i.c.) injection of tumor cells, the culture media was aspirated and cells were dislodged using 0.25% trypsin (Thermo Fisher). Trypsin was inactivated using a culture medium containing 10% FBS (Hyclone); then, the cell suspension was centrifuged to obtain a cell pellet, which was re-suspended in cell culture medium without serum to achieve a concentration of 1 × 10^5^ cells in 10 μL. Cell suspensions were kept at 2–4°C in an ice bath for no longer than 30 min. Male Sprague Dawley rats (Envigo, 400–500 g) were used to generate the 9L glioma model (*N* = 12). The top of the anesthetized rat’s head was shaved, fixed in a stereotaxic frame (Kopf-Tujunga), and the skull exposed via a midline incision. A burr hole was generated using a micro-drill with a 2.3 mm tip (CellPoint Scientific). A short beveled 26-gauge needle attached to the 50 μL Hamilton syringe (Hamilton Company), containing tumor cell suspension, was inserted into the brain −1.5 anterior-posterior, −4 mm lateral, −6 mm dorsal-ventral relative to bregma. The tumor cell suspension was slowly injected into the brain parenchyma over the period of 10 min to ensure steady resorption of injectate by the brain and to prevent the back-flux of cells into the subarachnoid and subdural spaces. After the needle was withdrawn, the hole in the dura was closed by cauterization, the burr hole filled with bone wax (Medline), and the skin incision closed using 3-0 black silk running suture (Ethicon). The rats were monitored post-operatively for signs of distress, weight loss, or neurological deficit and administered fluids (ie, saline by subcutaneous injection) or nutritional supplements, as needed.

### MR Imaging

T2-weighted MRI was performed 2 weeks following allograft implantation. The animals were anesthetized by inhalation of isoflurane (5% in oxygen for induction, and 2–2.5% for maintenance). During the imaging procedure, the animals were placed on a heated re-circulating water platform in order to maintain body temperature at 37^o^C. The animals were held in position using a bite bar and a home-built receive-only surface coil 2-element phased array was placed dorsal to the head, as described elsewhere.^47,48^ Images were acquired using a 7T ClinScan system (Bruker) operated by a Siemens console with Syngo software (Siemens). A localizing scan was performed and adjustments to the head position were made accordingly. Coronal and axial T2-weighted images were obtained (repetition time [TR] 3530 ms, echo time [TE] 38 ms, slice thickness 0.5 mm, field of view [FOV] 3.2 cm × 3.2 cm, resolution 125 µm × 125 µm × 1 mm, matrix 320 × 320). Images were processed using ImageJ software.

### PET Imaging Procedures in Animals

Baseline of 2-[^18^F]BzAHA PET/CT studies was performed a day after the MRI studies. The radiosynthesis and formulation of 2-[^18^F]BzAHA for intravenous (i.v.) injection was performed as previously described^45^; under inhalation anesthesia (as described above). Anesthetized rats were placed in a stereotactic head holder made of polycarbonate plastic (Kopf-Tujunga) and attached to the bed the microPET R4 scanner (Siemens) in the supine position with the long axis of the animal parallel to the long axis of the scanner and the brain positioned in the center of the FOV. The radiotracer (300–500 µCi/animal) was administered in saline via the tail vein in a total volume of ≤1.25 mL, as a slow bolus injection over the period of 1 min. Dynamic PET images were obtained over 60 min. After PET imaging, the positioning bed with the affixed anesthetized animal was transferred to the Inveon SPECT/CT scanner (Siemens) and CT images were acquired in 4 overlapping frames (2 min each) covering the whole body using X-ray tube settings of 80 kV and 500 µA with exposure time of 300–350 ms of each of the 360 rotational steps.

### Image Analysis and Quantification

Dynamic PET datasets were truncated into multiple 1–2 min static frames and images reconstructed using 2-dimensional ordered subsets expectation maximization (2D-OSEM) algorithm with 4 iterations and 16 subsets, as described before^43^; CT images were reconstructed using Shepp–Logan algorithm^49^; and PET/CT image fusion was accomplished using Inveon Research Workplace version 3.0 software package (Siemens). PET/CT and T2-weighted MR images of individual rat heads were manually co-registered using skull landmarks as fiducial markers in the AMIDE 1.0.4 software (http://amide.sourceforge.net). A digital rat brain atlas was used for the identification of specific structures of the brain and manual segmentation of T2-weighted MR images, based on stereotactic coordinates.^50^ Radioactivity concentration in specific brain structures was quantified with the AMIDE 1.0.4 software (http://amide.sourceforge.net) using regions of interest (ROI) analysis and expressed as µCi/g and standard uptake value (SUV). The SUV is defined as the ratio of the tissue radioactivity concentration (expressed as µCi/g tissue) at a given time point post-injection and the injected dose (in µCi, decay-corrected to the same time) and normalized by the body weight in grams. To obtain the ROI for the tumors on PET we used the MRI co-registration to identify the bulk of the tumor on PET. After identifying the bulk of the tumor on PET, the tumor area was outlined as an ROI and the upper quartile of voxel’s SUV was averaged as tumor SUV. These areas corresponded well with the histologic sections of tumor with increased SIRT1 expression. Time-activity curves (TACs) for different brain structures were plotted over time post-radiotracer administration. Logan graphical analysis with reference tissue^51^ was used for the quantitative analysis of the dynamic PET images with 2-[^18^F]BzAHA. The brainstem was used as a reference tissue due to the low levels of SIRT1 activity and subsequent low levels of 2-[^18^F]BzAHA-derived radioactivity accumulation in this region of the brain. The brainstem also receives similar levels of perfusion to the rest of the brain tissue thus providing a suitable reference tissue.

PET/CT/MRI with 2-[^18^F]BzAHA for monitoring the pharmacologic inhibition of SIRT1 expression–activity in intracerebral 9L gliomas in rats.Two days after initial 2-[^18^F]BzAHA PET/CT/MRI studies (baseline), rats bearing i.c. 9L gliomas were treated either with SIRT1-specific inhibitor EX-527 (5 mg/kg, i.p.; *N* = 3) or with HDAC class IIa inhibitor MC1568 (30 mg/kg, i.p.; *N* = 3) and 30 min later were injected i.v. with 300 μCi of 2-[^18^F]BzAHA. PET/CT images were acquired in dynamic mode over the period of 60 min. To facilitate comparative analyses, PET/CT/MR images acquired after EX-527 and MC1568 treatments were co-registered with pretreatment (baseline) images. 2-[^18^F]BzAHA SUVs before and after treatments with EX-527 or MC1568 were calculated for the same ROIs.

### Histology and Immunofluorescence Microscopy

Immediately following an imaging session, rats (*N* = 6) were anesthetized using sodium pentobarbital (50 mg/kg) and transcardially perfused with 4% formaldehyde in phosphate buffer. After fixation in 4% formaldehyde and 30% sucrose, coronal brain sections (20 µm) were obtained using OTF5000 cryomicrotome (Hacker–Bright Instruments) and set up as floating sections in buffered saline. For fluorescence immunohistochemistry of SIRT1, the sections were washed in PBS with triton detergent (PBS-T, 0.1M, pH 7.4) 3 × 3 min, followed by antigen retrieval at 70°C in sodium citrate buffer (pH 6.0) for 1 h, then washed in PBS-T 3 × 3 min. The sections were heated for 5 min at 55°C then washed with xylenes 3 times for 2 min each then rinsed in PBS 3 × 3 min. The sections were then incubated in 5% normal goat serum for 20 min at 20°C. Immediately following, the sections were washed 3 times in PBS-T and set up for 18-h incubation at 4°C in primary AlexaFluor488 conjugated anti-SIRT1 mouse monoclonal antibody (1:200, catalog number ab157401; Abcam). Following incubation, the antibody was removed and sections washed in PBS-T 3 times. The sections were mounted on high tissue-binding Superfrost Plus glass slides (Fischer Scientific) and coverslipped using an aqueous medium containing DAPI for nuclear stain (Vectashield; Vector Laboratories). Select sections were dual-immunostained using the rabbit anti-phospho-SIRT1 monoclonal antibody S47 (1:200, catalog number ab76039; Abcam) incubated for 14 h at 4°C, followed by 3 × 2 min washes in TBS and exposure to the secondary goat anti-rabbit polyclonal antibody conjugated to AlexaFluor647 (1:250, catalog number ab150079; Abcam) for 90 min at 4°C. Following 3 × 2 min washes in TBS the sections were mounted and coverslipped as described above. Fluorescence microscopy images were acquired using EVOS FL Auto (Life Technologies).

### Statistical Analyses

Excel 2016 (Microsoft) and Graph-Pad Prism 8 (GraphPad Software) were used for calculations and statistical analyses of data. Group data are reported as mean ± standard error. The differences in levels of radiotracer accumulation in tumors versus the contralateral brain structures were analyzed using one-way ANOVA. Drug-induced changes in the radiotracer uptake, SUV and distribution volume (DV), were analyzed using ANOVA for repeated measures. The *P* < .05 was considered as significant.

## Results

### 2-[^18^F]BzAHA PET/CT/MR Imaging of SIRT1 Expression–Activity of Intracerebral Gliomas in Rats

**Fig. 1:**
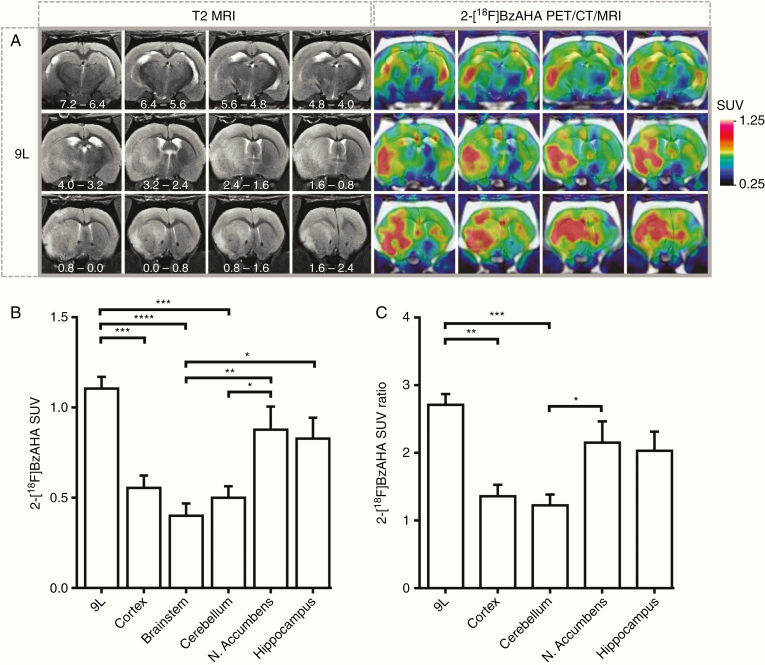
PET/CT/MRI of SIRT1 expression–activity in 9L intracerebral gliomas in rats. (A) Representative series of coronal images of the rat brain bearing intracerebral 9L glioma. The position of sections relative to bregma is indicated in millimeters on T2-weighted MR images. 2-[^18^F]BzAHA PET/CT images were obtained at 20 min post-injection of radiotracer and co-registered with T2-weighted MR images. The levels of 2-[^18^F]BzAHA accumulation in tumors and different structures of the brain were measured in standard uptake value (SUV). (B) SUV for 9L gliomas (*N* = 6) as compared to other structures within the brain. (C) SUV ratio normalized by the SUV of the brainstem region for 9L gliomas. PET/CT images are color coded to SUV. Data—mean ± SEM. Statistical significance was determined via one-way ANOVA, *P < .05, **P < .01, ***P < .001, ****P < .0001.

PET/CT/MRI with 2-[^18^F]BzAHA demonstrated heterogeneously increased, transient accumulation of 2-[^18^F]BzAHA-derived radioactivity in i.c. 9L tumors (Figure 1A; *N* = 6). The maximum contrast between tumors versus brainstem and cortex was observed at 15–20 min after i.v. administration of 2-[^18^F]BzAHA, resulting in SUVs of 1.11 ± 0.058 (Figure 1B) and tumor-to-brainstem SUV ratios of 2.73 ± 0.141 (Figure 1C) for 9L gliomas. Also, increased levels of retention of 2-[^18^F]BzAHA-derived radioactivity were observed in normal structures of the brain that are known to express higher levels of SIRT1, including *hippocampus* and *nucleus accumbens*. Similar magnitudes of 2-[^18^F]BzAHA TACs were observed during the first few minutes after i.v. administration in normal brain structures expressing high levels of SIRT1 (i.e., *nucleus accumbens* and *hippocampus*) and the magnitudes of TACs observed in 9L gliomas (Supplementary Figure S1).

### Histological and Immunofluorescence Analyses of 9L Brain Tissue Sections

**Fig. 2:**
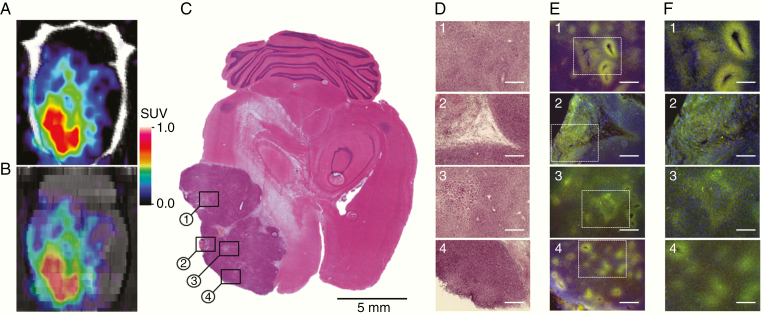
Validation of 2-[^18^F]BzAHA PET/CT/MRI using histopathologic and immunofluorescence (IF) analyses of 9L glioma. (A) 2-[^18^F]BzAHA axial PET/CT image (top), (B) axial 2-[^18^F]BzAHA PET/MRI fusion image (bottom), PET/CT images are color-coded to standard uptake values (SUVs). (C) Macroscopic and (D) microscopic images (10× objective, scale bar—200 µm), of axial brain sections stained with H&E to visualize tumor structure. Microscopic images of SIRT1 IF and DAPI (E) 10x objective, scale bar—200 µm; (F) 20x objective, scale bar—100 µm.

To validate the results of noninvasive PET/CT/MRI of SIRT1 expression–activity and to determine which tumor regions contributed to increased 2-[^18^F]BzAHA uptake, 6 animals bearing 9L tumors underwent perfusion fixation immediately after the imaging session. Their brains were extracted for histologic analyses. H&E staining of brain tissue sections confirmed the localization of tumors observed on MRI and PET images (Figure 2A–C). Immunofluorescence staining (IF) in 9L tumors for SIRT1 demonstrated that the enzyme is overexpressed in the tumor parenchyma in a heterogeneous pattern. Histopathological features typical to gliomas display increased SIRT1 IF, particularly in hypoxic areas (ie, regions of microvessel proliferation, pseudopalisades, and peri-necrotic areas). Glioma cells surrounding cavernous vessels (Figure 2D–F, *top row*) as well as in peri-necrotic glioma regions (Figure 2D–F, *second row*) observed on H&E show higher SIRT1 expression, as compared to non-hypoxic or less hypoxic-appearing surrounding tumor regions. In particular, the upregulation of SIRT1 expression was observed in pseudopalisading zones (Figure 2D–F, *third row*). Also, multiple foci of SIRT1 expression are dispersed throughout the tumor parenchyma (Figure 2D–F, *bottom row*).

**Fig. 3:**
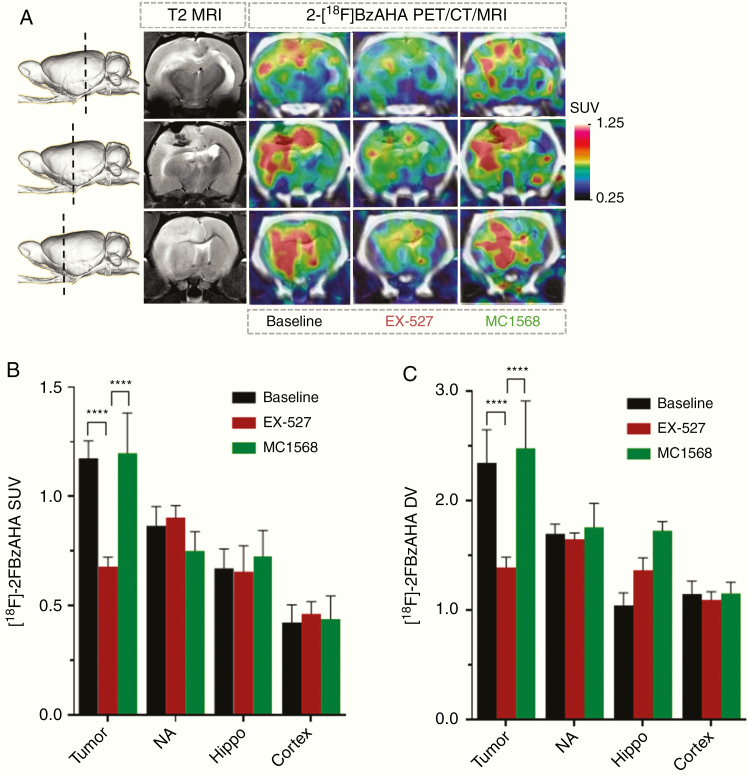
Monitoring pharmacologic inhibition of SIRT1 with 2-[^18^F]BzAHA PET/CT/MRI. (A) Representative coronal T2-weighted MRI and 2-[^18^F]BzAHA PET/CT/MR images depicting different regions of the rat brain with intracerebral 9L tumor (shown by a dotted line on a side-view of a 3D rendered image): through the area of the *hippocampus* (Hippo, top), 9L tumor (9L, middle), *nucleus accumbens* (NA, bottom). 2-[^18^F]BzAHA PET/CT images were obtained at 20 min post-radiotracer administration before (baseline) and after treatment with either EX-527 (SIRT1 selective inhibitor) or MC1568 (HDAC class IIa selective inhibitor). PET/CT images are color-coded to standard uptake values (SUVs). The levels of 2-[^18^F]BzAHA-derived radioactivity are expressed as (B) SUVs or (C) distribution volumes (DVs) in 9L tumors and different brain structures at baseline (*N* = 4) and after therapy with EX-527 (*N* = 3) or MC1568 (*N* = 3). Data—mean ± SEM. Statistical significance was determined using ANOVA for repeated measures; ****P < .0001.

PET/CT/MR imaging with 2-[^18^F]BzAHA for monitoring the pharmacologic inhibition of SIRT1 expression–activity in intracerebral 9L gliomas in rats.Treatment of rats (*N* = 3) with a single dose of EX-527 (5 mg/kg, i.p., 30 min prior to injection of 2-[^18^F]BzAHA) resulted in the inhibition of SIRT1 activity in 9L tumors, as evidenced by statistically significant decreases in SUV (*P* < .005) and DV (*P* < .005) of 2-[^18^F]BzAHA as compared to baseline levels (Figure 3; Supplementary Figure S2). However, EX-527 treatment did not significantly reduce the SUV and DV of 2-[^18^F]BzAHA in the contralateral *nucleus accumbens*, *hippocampus*, or cortex. Treatment of rats (*N* = 3) with a single dose of MC1568 (30 mg/kg, i.p., 30 min prior to injection of 2-[^18^F]BzAHA) did not inhibit SIRT1 expression–activity neither in 9L, nor in contralateral brain structures, as evidenced by the lack of statistically significant decreases in 2-[^18^F]BzAHA SUV and DV, as compared to baseline levels (Figure 3; Supplementary Figure S3).

## Discussion

Current studies demonstrated that the utility of PET/CT/MRI with 2-[^18^F]BzAHA for quantitative imaging of SIRT1 expression–activity is well-established and characterized in i.c. 9L allograft models in rats and expanded our previous studies on imaging SIRT1 expression–activity in the normal brain.^45,52^ The level of expression–activity of SIRT1 in 9L gliomas was significantly higher than in the brainstem, contralateral cortex, cerebellum, *nucleus accumbens*, and *hippocampus*, as evidenced by both SUV and DV of 2-[^18^F]BzAHA. The imaging findings in normal structures of the brain are consistent with our previous studies on imaging SIRT1 expression–activity levels.^45^

The spatial distribution of HDAC class IIa expression-activity in 9L gliomas was heterogeneous, as visualized using 2-[^18^F]BzAHA PET/CT/MRI and validated by IF microscopy of brain and tumor tissue sections. Significantly higher levels of SIRT1 expression were observed in hypoxic peri-necrotic tumor regions, as compared to non-hypoxic or less hypoxic-appearing surrounding tumor regions. In 9L tumors, increased levels of SIRT1 expression were observed in tumor cells surrounding cavernous vessels and in the pseudopalisading zones. Multiple foci of SIRT1 over-expression were dispersed throughout the i.c. tumor parenchyma, demonstrating tumor cell subpopulations responding to hypoxia and other local stressors through epigenetic mechanisms. These observations are consistent with the known roles of SIRT1 in epigenetic adaptive mechanisms to hypoxic/hyponutrient stress.^53–55^ SIRT1 deacetylates and stabilizes HIF-1α,^25^ which increases the expression of VEGF, GLUT1, and MMP2^25^. Nutritional and metabolic stress in tumor cells causes activation of AMP-activated protein kinase, which in turn activates SIRT1 by inducing the dissociation of SIRT1 from its endogenous inhibitor, deleted in breast cancer 1.^56^ Increased expression–activity of SIRT1 under hyponutrient and/or hypoxic stress promotes cell survival, proliferation, and angiogenesis and inhibits apoptosis.^26–32^ Also, SIRT1 and other members of HDAC class III family of protein deacetylases play important roles in molecular mechanisms of DNA repair induced by chemo- and radiation therapy.^57,58^ The chemo- and radioresistance of glioblastoma stem cell clones are associated with metabolic adaptation to reduced glucose dependence mediated by the upregulation of SIRT1–PGC-1α axis and DNA repair genes,^36^ while SIRT1 knockdown inhibits glioma cell proliferation and potentiates TMZ toxicity via facilitation of reactive oxygen species generation.^59^ Therefore, SIRT1 represents a potential target for the development of novel approaches to therapy of gliomas and other tumor types overexpressing SIRT1.

However, currently there is a controversy in the published literature regarding the levels of expression versus prognostic and mechanistic roles of SIRT1 in gliomas and GBM, in particular. While some clinical studies report that majority of gliomas express increased levels of SIRT1 (both mRNA and protein) which are predictive of poor prognosis, as compared to gliomas expressing lower levels of SIRT1.^59,60^ In contrast, other studies report reduced levels of SIRT1 expression in gliomas,^61^ that 80% of GBM tumors exhibit a loss of one allele of the gene encoding SIRT1,^62^ and that decreased levels of SIRT1 (both mRNA and protein) are predictive of poor prognosis, as compared to GBMs with diploid SIRT1, expressing higher levels of SIRT1. Potential reasons to this controversy may be partly due to spatial heterogeneity of gliomas (especially GBM) and variability of biopsy sites and tissue sampling during neurosurgical resection of gliomas in different clinical studies (ie, from the infiltrating edge versus hypoxic and/or necrotic core). This controversy may have developed because quantitative measurements of SIRT1 mRNA expression levels do not necessarily reflect the levels of SIRT1 protein expression, which is dependent not only on transcriptional regulation, but mostly on post-transcriptional factors (ie, different miRs). Also, the levels of total SIRT1 protein expression do not necessarily reflect the level of SIRT1 enzyme activity, which are regulated by multiple post-translational modifications of SIRT1 protein (ie, phosphorylation, ubiquitination, sumoylation, carbonylation, etc.) and the availability of critical metabolic cofactors, such as NAD^+^.^63^ Noninvasive in vivo imaging of SIRT1 expression–activity in gliomas using PET/CT/MRI with 2-[^18^F]BzAHA may help to resolve this controversy by providing the means for the initial molecular profiling, image guidance of biopsies, and for image-guided resection of glioma samples for further molecular genetic analyses. The role of SIRT1 expression–activity on progression and prognosis of gliomas can now be investigated more accurately using quantitative imaging of SIRT1 expression–activity in tumor tissue in vivo, in the native microenvironment of individual tumors.

Previous studies in patient-derived glioblastoma cells demonstrated that SAHA, VPA, MS275, LBH589, and Scriptaid are effective radiosensitizers of gliomas.^64^ However, the potential efficacy of SIRT1-specific pharmacologic inhibitors, such as EX-527 (Selisistat),^65^ for treatment of brain gliomas has not been investigated in preclinical studies or in clinical trials. In this study, we demonstrated the potential utility of PET/CT/MRI with 2-[^18^F]BzAHA for quantitative monitoring of the EX-527-induced pharmacologic inhibition of SIRT1 expression–activity in i.c. 9L tumor xenografts in rats. EX-527 administered in rats as a single dose of 5 mg/kg i.p., 30 min prior to injection of 2-[^18^F]BzAHA caused almost a 2-fold inhibition of SIRT1 expression–activity in i.c. 9L gliomas, as evidenced by the comparison of baseline to posttreatment SUV and DV of 2-[^18^F]BzAHA. Pretreatment of 9L tumor-bearing rats with EX-527 at this dose did not significantly reduce the magnitude of SIRT1 expression–activity in the contralateral brain structures (ie, nucleus accumbens, hippocampus) that are known to overexpress SIRT1.^45,66–69^ Such differences in the magnitude of SIRT1 inhibition in 9L gliomas versus contralateral brain structures can be explained, at least in part, by higher concentrations of EX-527 delivered to the 9L tumor tissue through the leaky tumor microvasculature, as compared to relatively lower concentrations of EX-527 delivered in the contralateral brain structures with intact blood–brain barrier (BBB). Considering the mechanism of action of EX-527, it is unlikely that pretreatment with EX-527 by i.p. route 30 min prior to injection of 2-[^18^F]BzAHA could have caused an acute reduction (normalization) of BBB permeability in the 9L tumors, which could explain the reduction in the apparent DV of 2-[^18^F]BzAHA-derived radioactivity. Moreover, high lipophilicity of 2-[^18^F]BzAHA (LogP = 4.83) enables it to diffuse across the capillaries with intact BBB and rapidly equilibrate between blood and tissue compartments in the normal brain regions.^45^ The latter is evidenced by the high level of 2-[^18^F]BzAHA in the normal brain observed with PET during the first few minutes after i.v. administration and higher SUVs and DVs of 2-[^18^F]BzAHA-derived radioactivity in the brain structures expressing high levels of SIRT1. However, using a single tracer, such as 2-[^18^F]BzAHA, it is hard to determine the degree to which a single dose of EX-527 may influence BBB permeability or perfusion and, thus, the delivery of 2-[^18^F]BzAHA in 9L gliomas. This could be studied using multi-tracer approaches or dynamic contrast-enhanced MRI. The dose of EX-527 (5 mg/kg i.p.) used in this study was chosen to be similar to doses used in our initial studies with 2-[^18^F]BzAHA in the normal brain^45^ and previously published reports.^65^ However, this dose caused only about 40% inhibition of SIRT1 expression–activity and 2-[^18^F]BzAHA-derived radioactivity accumulation in 9L tumors and only insignificant changes in SIRT1 activity in the contralateral brain structures. This observation suggests that higher doses of EX-527 may be required to achieve a more complete inhibition of SIRT1 in 9L tumors. Using PET/MRI with 2-[^18^F]BzAHA it should be possible to determine the IC_50_ doses of EX-527 and other SIRT1-specific inhibitors or activators in gliomas and different structures of the brain, as reported by us previously for monitoring SAHA-induced inhibition of HDACs class IIa in the primate brain using PET/CT/MRI with [^18^F]TFAHA.^41^ The observed inhibition of SIRT1 expression–activity in 9L tumors by EX-527 was isoform-selective, as evidenced by the lack of SIRT1 inhibition by pretreatment with MC1568, which is the HDACs class IIa selective inhibitor.^70^ These findings extend our initial studies in the normal rat brain using PET/CT/MRI with 2-[^18^F]BzAHA for monitoring the EX-527-induced pharmacologic inhibition of SIRT1 expression–activity in normal brain structures known to overexpress SIRT1 (ie, hippocampus, amygdala, arcuate nucleus, nucleus accumbens).^45^ Also, these findings are consistent with results of our studies using PET/CT/MRI with [^18^F]TFAHA that demonstrated effective inhibition of HDACs class IIa expression–activity in i.c. 9L tumors by MC1568, and the lack of inhibition of HDACs class IIa expression–activity by EX-527.^44^

It has been previously reported that many tumor-suppressor microRNAs (miRs) are down-regulated in gliomas, leading to aberrant gene expression and glioma progression.^71^ Administration of exogenous miR has been tested for treatment of gliomas, including miR-22, miR-34a, miR-132, miR-133b, miR-181a, miR-200a, miR-217, miR-320, and miR-3908.^72–79^ The MRX34 (miR-34a mimic) has been tested in a phase I multicenter clinical trial (NCT01829971) for the treatment of advanced solid tumors and demonstrated evidence of antitumor activity in a subset of patients.^80^ PET/CT/MRI with 2-[^18^F]BzAHA could be potentially utilized for selection of glioma patients with high levels of SIRT1 expression–activity levels in tumor tissue that may benefit from SIRT1-targeted pharmacologic and genetic therapies, including miR mimics, for monitoring the pharmacodynamics and dose-dependency of SIRT1 inhibition and prediction of treatment response.

## Conclusions

The current study demonstrated that PET/CT/MR imaging with 2-[^18^F]BzAHA allows for noninvasive and repetitive quantification of spatial localization and temporal dynamics of SIRT1 expression–activity in i.c. tumors before and after treatment with SIRT1-specific inhibitor EX-527. PET/CT/MR imaging with 2-[^18^F]BzAHA may facilitate future clinical studies aimed to elucidate the role of the SIRT1 enzyme in gliomagenesis and progression, and to optimize therapeutic doses of novel SIRT1 inhibitors in combined chemo- and radiotherapy of GBM.

## Supplementary Material

vdaa006_suppl_Supplementary_Figure_S1

vdaa006_suppl_Supplementary_Figure_S2

vdaa006_suppl_Supplementary_Figure_S3

vdaa006_suppl_Supplementary_Figure_Legends
