# Supplementary figures and images for: Noninvasive quantification of SIRT1 expression–activity and pharmacologic inhibition in a rat model of intracerebral glioma using 2-[18F]BzAHA PET/CT/MRI
Source: Neurooncol Adv. 2020 Jan 16;2(1):vdaa006. doi: 10.1093/noajnl/vdaa006 (PMC7034639; doi:10.1093/noajnl/vdaa006)

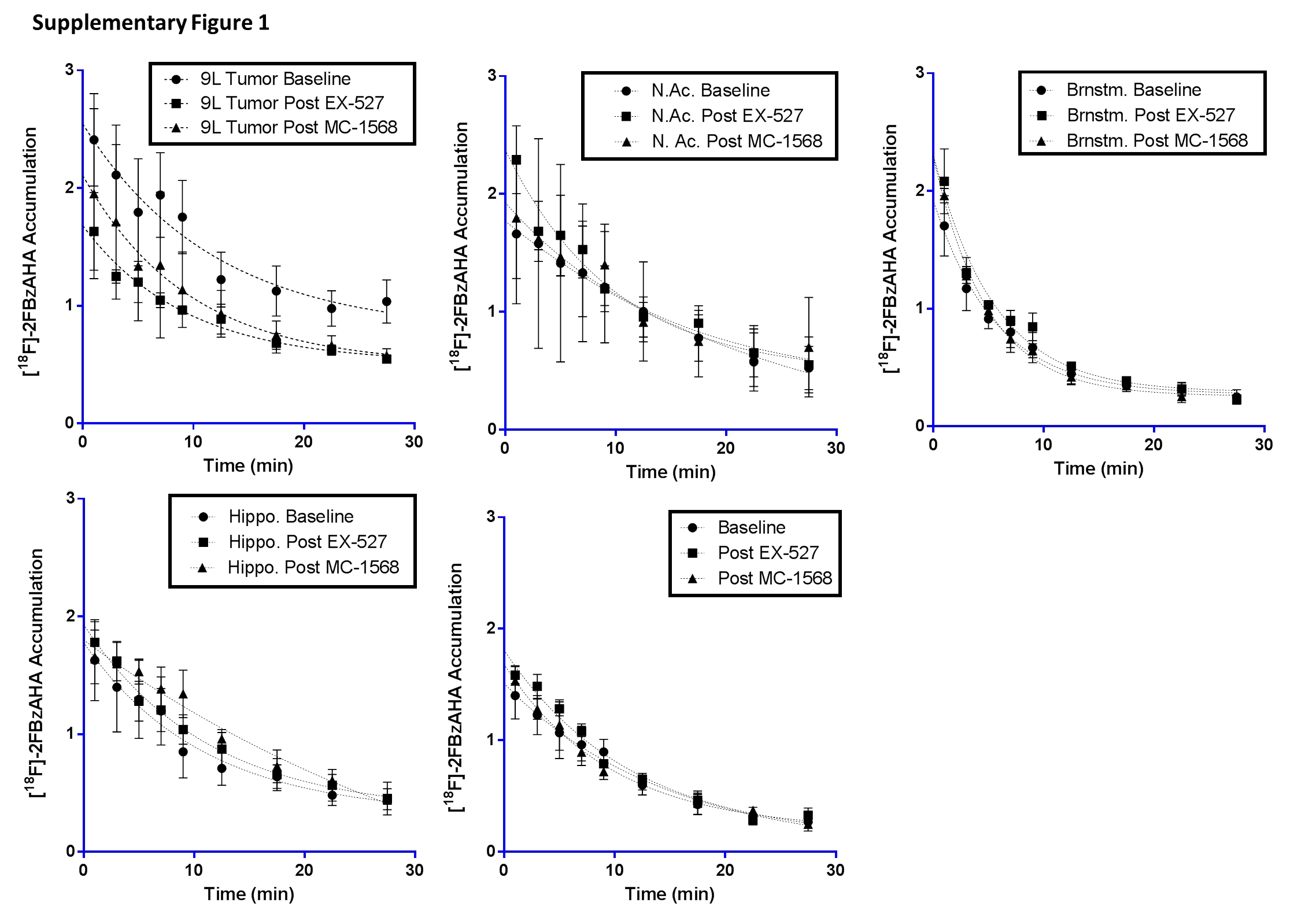

Supplement: vdaa006_suppl_Supplementary_Figure_S1 [file vdaa006_suppl_supplementary_figure_s1.png]

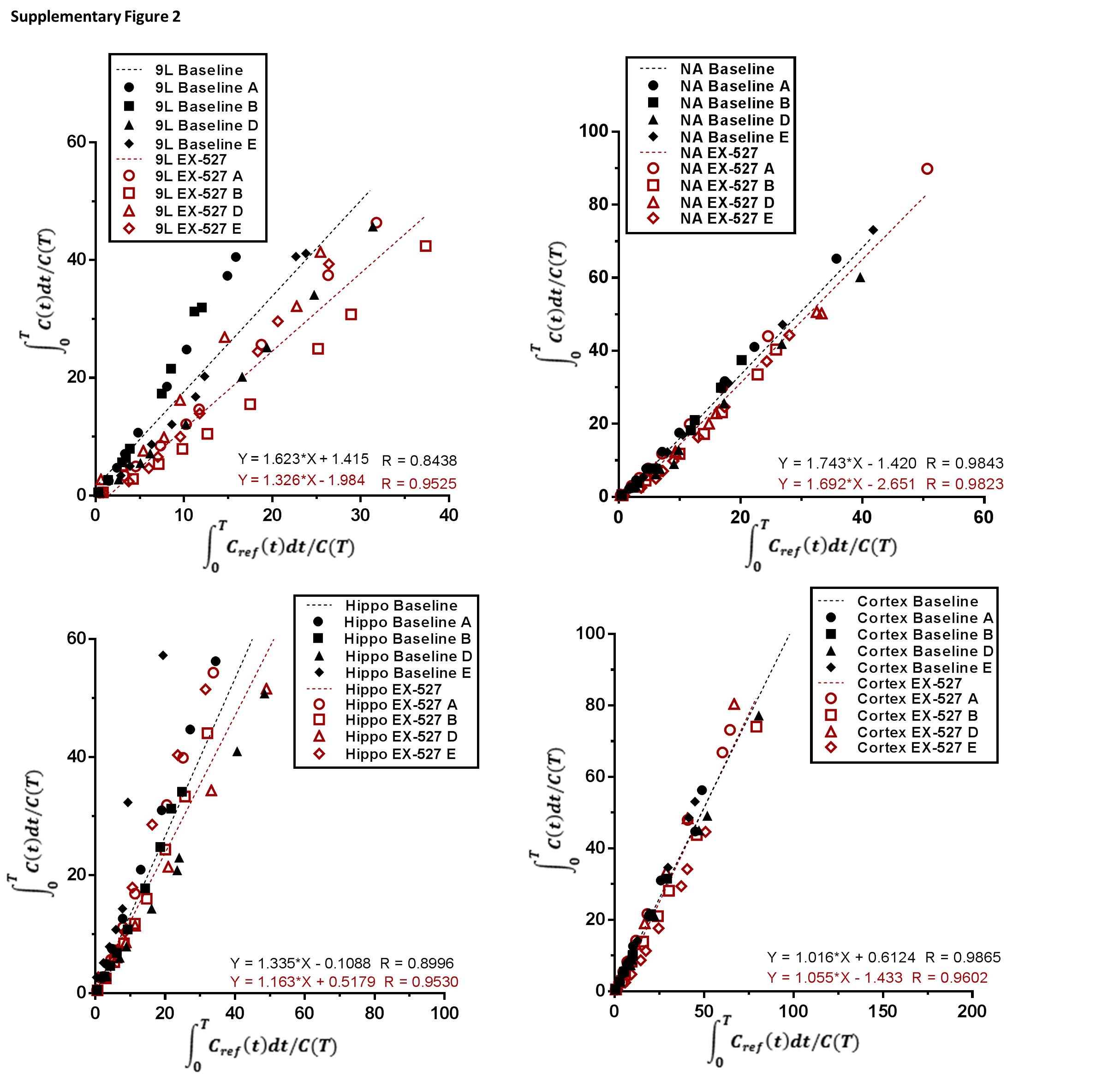

Supplement: vdaa006_suppl_Supplementary_Figure_S2 [file vdaa006_suppl_supplementary_figure_s2.png]

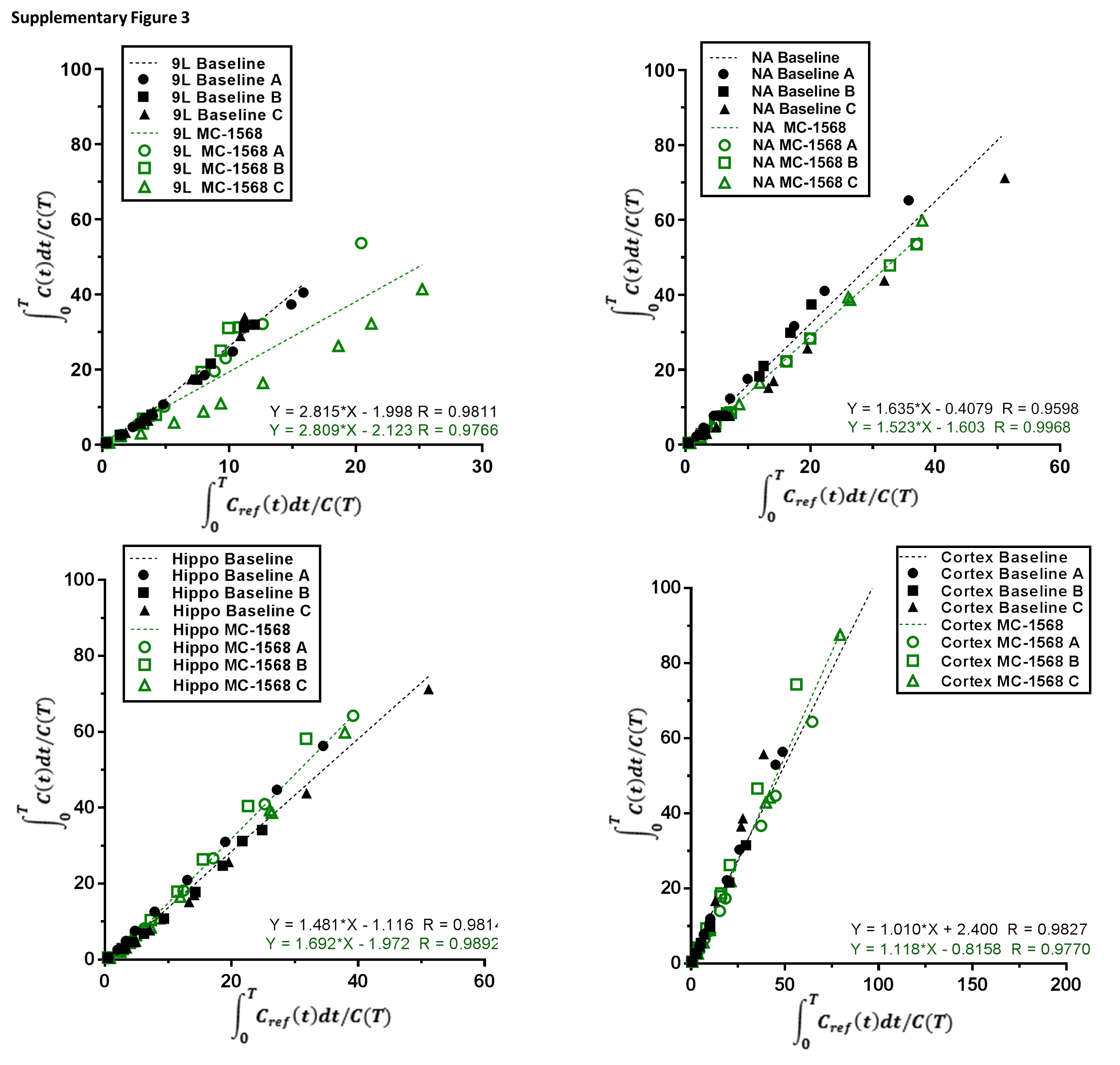

Supplement: vdaa006_suppl_Supplementary_Figure_S3 [file vdaa006_suppl_supplementary_figure_s3.png]
