# Supplementary material for: Noninvasive quantification of SIRT1 expression–activity and pharmacologic inhibition in a rat model of intracerebral glioma using 2-[18F]BzAHA PET/CT/MRI
Source: Neurooncol Adv. 2020 Jan 16;2(1):vdaa006. doi: 10.1093/noajnl/vdaa006 (PMC7034639; doi:10.1093/noajnl/vdaa006)
Supplement: vdaa006_suppl_Supplementary_Figure_Legends [file vdaa006_suppl_supplementary_figure_legends.docx]

**Figure S1.** Time-activity curves of 2-[^18^F]BzAHA after intravenous administration in the brain of rats bearing intracerebral 9L gliomas: **A**) 9L gliomas; **B**) white matter; **B**) *n.accumbens* (N. Ac.); **C**) brainstem; **D**) *hippocampus* (Hippo); **F**) cortex. Data points in the time-activity curves determined under different conditions are: before treatment – circles; after treatment with EX-527 – squares; after treatment with MC1568 – triangles. Data points: mean±SEM. The magnitude of 2-[^18^F]BzAHA-derived radioactivity in individual regions is presented as standard uptake value (SUV). Time scale is in minutes (min). The magnitude of 2-[^18^F]BzAHA time-activity curves (TACs) was statistically significantly decreased (p<0.005) in 9L gliomas after pre-treatment of animals with EX-527, whereas no significant differences in the magnitude and profiles of 2-[^18^F]BzAHA TACs were observed after pre-treatment with EX-527 (**Fig. S1**). Also, no statistically significant decreases in 2-[^18^F]BzAHA TACs were observed in the contralateral normal brain structures known to overexpress SIRT1 (i.e., n.accumbens and *hippocampus*).

**Figure S2.** Logan graphical analyses of transient retention of 2-[^18^F]BzAHA-derived radioactivity in 9L tumors and different brain structures in the same animals (N=3) at baseline (black) and after therapy with EX-527 (red); data points for individual animals are shown in different shapes (circle, square, triangle). Panels: 9L (9L glioma), Hippo (contralateral *hippocampus*), NA (contralateral *n.accumbens*), and Cortex (contralateral cerebral cortex). Linear regression equations for individual animal data points are listed in the same sequence as the data legends. Linear regression coefficients represent distribution volumes of 2-[^18^F]BzAHA-derived radioactivity in 9L tumors and various structures of the brain.

**Figure S3.** Logan graphical analyses of transient retention of 2-[^18^F]BzAHA-derived radioactivity in 9L tumors and different brain structures in the same animals (N=3) at baseline (black) and after therapy with MC1568 (green); data points for individual animals are shown in different shapes (circle, square, triangle). Panels: 9L (9L glioma), Hippo (contralateral *hippocampus*), NA (contralateral *n.accumbens*), and Cortex (contralateral cerebral cortex). Linear regression equations for individual animal data points are listed in the same sequence as the data legends. Linear regression coefficients represent distribution volumes of 2-[^18^F]BzAHA-derived radioactivity in 9L tumors and various structures of the brain.
